# Interferon regulatory factor 4 binding protein is a novel p53 target gene and suppresses cisplatin-induced apoptosis of breast cancer cells

**DOI:** 10.1186/1476-4598-11-54

**Published:** 2012-08-13

**Authors:** Mingzhen Yang, Fang Yuan, Peng Li, Zhongjiao Chen, An Chen, Shuhui Li, Chuanmin Hu

**Affiliations:** 1 Department of Clinical Biochemistry, Third Military Medical University, Chongqing, 400038, P.R. China; 2 Department of Cell Biology, Third Military Medical University, Chongqing, 400038, P.R. China; 3 Urology Institute of People’s Liberation Army, Southwest Hospital, Third Military Medical University, Chongqing, 400038, P.R. China

**Keywords:** Breast cancer, Interferon regulatory factor 4 binding protein (IBP), p53, Cisplatin, Apoptosis

## Abstract

**Background:**

Our previous work demonstrated that ectopic expression of interferon regulatory factor 4 binding protein (IBP) was correlated with the malignant behaviour of human breast cancer cells. The mechanisms controlling differential expression of IBP in breast cancer still remain unknown.

**Results:**

To investigate the mechanism of IBP dysregulation in breast cancer, we identified IBP was a novel p53 target gene. IBP expression was negatively regulated by wild-type p53 and was p53 dependently suppressed by DNA damage agent cisplatin. Furthermore, high levels of IBP were found to decrease cisplatin-induced growth suppression and apoptotic cell death, which was associated with decreased p53 activity and imbalanced Bcl-2 family member expression.

**Conclusions:**

IBP is a novel p53 target gene which suppresses cisplatin-mediated apoptosis of breast cancer cells *via* negative feedback regulation of the p53 signalling pathway, suggesting IBP may serve as a target for pharmacologic intervention of breast cancer resistant to cisplatin therapy.

## Background

Since its discovery over 30 years ago, p53 has been shown to play a key role in mediating cell responses to stress. p53 primarily accomplishes this by inducing or repressing a number of genes involved in cell cycle arrest, senescence, apoptosis, DNA repair, and angiogenesis [1]. Among the roles of p53, its tumor-suppression activity is associated with its ability to function as a transcriptional master regulator [2]. The identification of additional p53 target genes is steadily progressing and may elucidate the mechanisms by which p53 exerts its tumour-suppression activity.

Breast cancer is the most frequent cancer in women. An estimated 1.15 million new cases of breast cancer were identified in 2002. In China, breast cancer registries record annual incidence increases of 3% to 4% [3]. Genetic studies have revealed that at least one third of nonfamilial breast cancers contain mutations in p53 [4], and 1,400 p53 mutations have been identified in breast cancer [5]. Efficacy of p53 activity represents a vulnerable link in the barriers to tumorigenesis in the breast epithelium [6]. In addition to its role in tumorigenesis, p53 also affects the effect of platinum therapy [7]. Previous studies have shown that the p53 pathway is inactivated in cisplatin-resistant MCF-7 breast cancer cells [8].

The Interferon regulatory factor 4 binding protein (IBP) gene (NM_022047), also known as DEF6 or SLAT, has been mapped to human chromosome 6p21.31 and is centromeric to the MHC locus [9]. IBP is broadly expressed in immune cells and can be detected in both T- and B-cell compartments. In the immune system, IBP functions as a guanine nucleotide exchange factor (GEF), which is an upstream activator of the Rho-family GTPases activates the Rac1, RhoA and CDC42 GTPases [10,11], modulates TCR-induced signalling events [12], and regulates TLR4-mediated signalling [13]. Loss of IBP in mice led to the spontaneous development of systemic autoimmunity [14]. Studies have shown that IBP has functions in other systems. IBP is expressed in muscle cells and influences myoblast differentiation [11]. It is one of the top five genes that distinguish extraskeletal myxoid chondrosarcoma (EMC) from other sarcomas [15]. Our laboratory reported that IBP was over-expressed in a considerable proportion of human breast and colorectal cancers [16,17]. IBP and p53 protein levels were negatively correlated among 107 breast cancer tissue samples [16]. The expression pattern of IBP, its transcriptional regulation, and especially the link between IBP and p53 in breast cancer are poorly understood.

In the present study, we sought to better understand the mechanisms controlling differential expression of IBP. We found that IBP contains a noncanonical p53-binding site in its 5′-flanking region. IBP expression was suppressed when wild-type p53 was directly bound to IBP promoter. Further, IBP was down-regulated by the DNA damage agents in breast cancer cell lines. Breast cancer cells overexpressing IBP were resistant to cisplatin-induced growth suppression and apoptosis. IBP knockdown increased cisplatin chemosensitivity and up-regulated p53 expression. Our results demonstrate that IBP is a novel p53 target gene which suppresses cisplatin-mediated apoptosis of breast cancer cells *via* negative feedback regulation of the p53 signaling pathway.

## Results

### p53 inhibits the transcriptional activity of the IBP promoter

**Figure 1:**
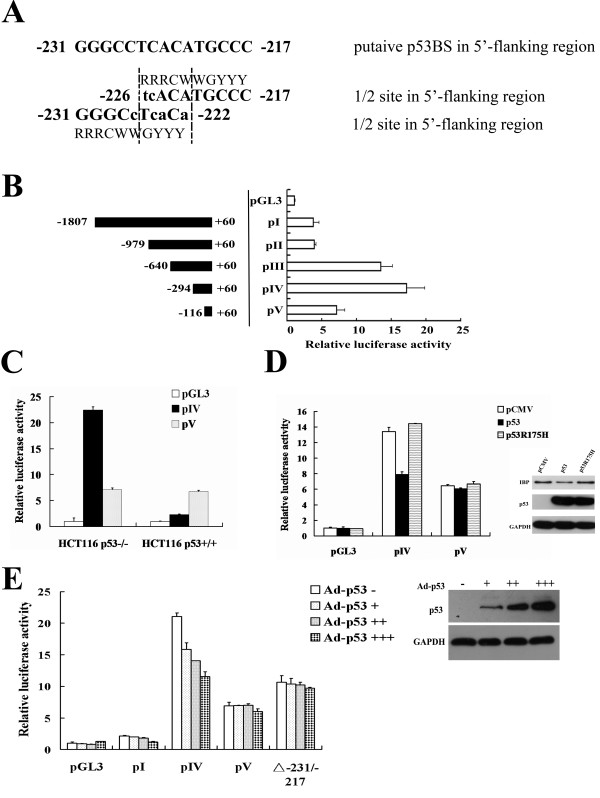
**p53 inhibits the transcriptional activity of the IBP promoter. (A)** The putative p53-binding site that is located in the promoter region of the IBP gene is shown. The −231 to −222 and −226 to −217 regions contain noncanonical 1/2 sites. **(B)** Schematic representations of the five promoter-reporter constructs that contained different-length fragments that were cloned into pGL3-basic vector are shown. The promoter-luciferase constructs or the promoterless vector was transiently transfected into HEK293 cells. The luciferase activity was normalised to *Renilla* luciferase activity that was expressed by pRL-TK and is presented as the mean ± SD of triplicate experiments. **(C)** HCT116 p53^−/−^ and HCT116 p53^+/+^ cells were transfected with pIV, pV or pGL3-basic respectively. **(D)** IBP promoter construct pIV or pV was cotransfected with pCMV-p53 or pCMV-p53R175H into HCT116 p53^−/−^ cells. The cotransfection with the empty pCMV vector serves as a control. The right inset shows the expression of IBP and p53 in infected HCT116 p53^−/−^ cells. **(E)** HCT116 p53^−/−^ cells were infected with Ad-p53 in the progressively increased concentration. (+, 10^2^; ++, 10^4^; +++,10^6^ plague-forming units). The right inset shows the expression of p53 in infected HCT116 p53^−/−^ cells. Cells were transfected with the IBP promoter constructs (pI, pIV, pV or −231/-217 deleted pIV).

To investigate transcriptional regulation of IBP, we first analyzed the 5′-flanking region of IBP gene. PROMO bioinformatics analysis (http://alggen.lsi.upc.edu/) [18,19] demonstrated that it contained two p53 binding sequences: −231 to −225 (GGGCCTC) and −223 to −217 (CATGCCC). The canonical p53-binding site was originally defined as RRRCWWGYYY and contained a separation of 0 to 13 bp, where R = purine, Y = pyrimidine, and W = A or T [20]. The noncanonical sequences were composed of 3/4 or 1/2 sites that are functional targets for p53 transactivation [2,21]. As shown in Figure 1A, the IBP gene −231 to −217 contained a putative noncanonical p53-binding site with a 1/2 site. To examine whether the putative IBP p53-binding site was functionally responsible for p53-dependent transcription, we subcloned 5′-deletion mutants of the IBP 5′-flanking region into a luciferase expression vector pGL3-basic, and fragment pIV (−294 to +60), which has the strongest transcriptional activity (Figure 1B) and harbours p53-binding site, was transiently transfected into HCT116 p53^−/−^ or HCT116 p53^+/+^ (wild-type p53) cells. pIV exhibited higher luciferase activity in p53 knockout HCT116 cells (Figure 1C). When pIV or pV was co-transfected with an empty pCMV, pCMV-p53 or pCMV-p53R175H vector into p53 null HCT116 cells, pCMV-p53 significantly decreased the luciferase activity of pIV. pCMV-p53R175H, which expressed a p53 mutant, did not affect pIV luciferase activity (Figure 1D). Additionally, we infected HCT116 p53^−/−^ cells with Ad-p53 at increasing concentrations. pIV exhibited a dose dependent luciferase activity decrease in response to increased Ad-p53, while pV did not. And when the putative p53-binding site (−231/-217) was deleted from pIV, Ad-p53 did not significantly decrease the luciferase activity (Figure 1E). These observations indicate that functional p53 decreases the activity of the IBP promoter through its putative p53-binding site.

### p53 attenuates IBP expression

**Figure 2:**
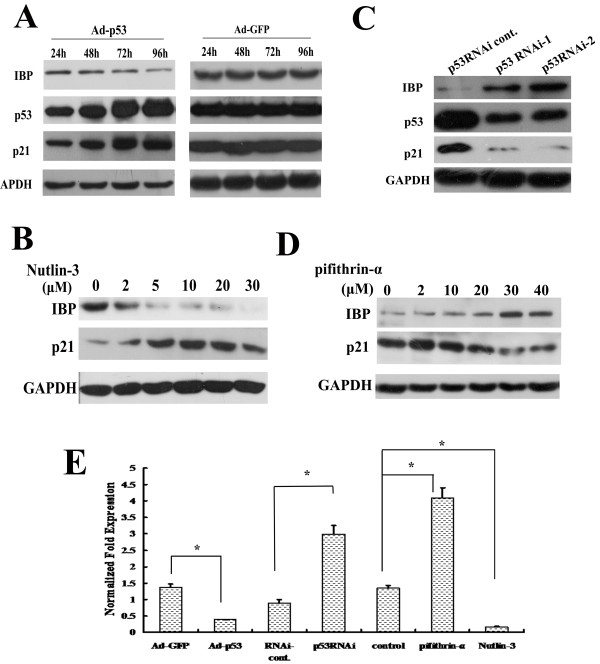
**p53 attenuates IBP expression.(A** and **C)** The expression of IBP and p53 in MCF-7 cells were tested by Western blot after Ad-p53 infection at different time points **(A)** or p53 RNAi lentiviruses infection **(C)**. Ad-GFP or p53 RNAi cont. was a respective control. **(B and D)** The expression of IBP was detected by Western blot in MCF-7 cells treated with different concentrations of Nutlin-3 **(B)** or pifithrin-α **(D)**. GAPDH and p21 served as controls. **(E)** Real-time PCR analysis of the expression of IBP in MCF-7 cells treated with Ad-p53 (96 h), p53-RNAi, Nutlin-3(10 μmol/L for 8 h ) or pifithrin-α (30 μmol/L for 24 h); GAPDH was used as a control. * , p < 0.01.

To further test whether p53 decreases IBP expression, MCF-7 cells (wild-type p53) were infected with Ad-p53 or Ad-GFP (as a control). After 96 h IBP protein was significantly decreased with increased p53 expression (Figure 2A). To determine the effects of endogenous p53 on IBP expression, we treated MCF-7 cells with MDM2 antagonist Nutlin-3 [22] for 8 h. The IBP protein level was dose-dependently attenuated (Figure 2B). And in p53 null HCT116 cells, Nutlin-3 could not decrease IBP expression (see Additional file 1). To determine whether p53 was required for IBP suppression, p53-targeting RNAi lentiviral particles and the p53 inhibitor pifithrin-α [23] were used in MCF-7 cells. The knockdown of p53 in MCF-7 cells increased IBP expression (Figure 2C), and an increased IBP protein expression was observed with increasing doses of pifithrin-α (Figure 2D). p21, which is a p53-responsive gene [24], was used as an internal control in these experiments. To test whether p53 regulates transcriptional level of IBP, quantitative RT-PCR was performed. As shown in Figure 2E, Ad-p53 and Nutlin-3 decreased IBP expression, while pifithrin-α and p53-targeting RNAi lentiviral particles increased IBP expression. These results indicate that IBP expression is directly associated with p53 activation and thus is a p53-responsive gene.

### p53 protein binds to IBP core promoter

**Figure 3:**
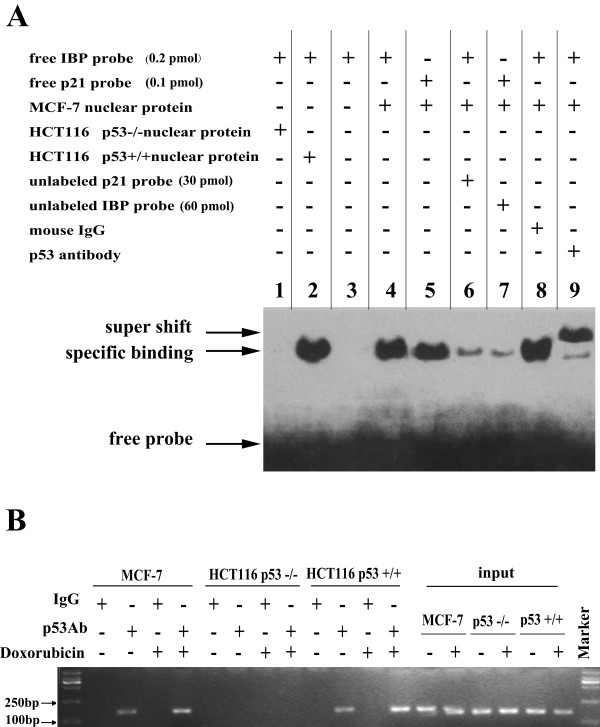
**Recruitment of the p53 protein to the IBP promoter. (A)** EMSA assay demonstrating the ability of p53 to bind the IBP promoter *in vitro.* The binding reactions were performed with nuclear extracts and various labelled oligonucleotides in the presence of unlabelled probes or antibodies as indicated. HCT116 p53^−/−^ nuclear proteins were extracted as a negative control. A p21 probe served as a positive control. The band was supershifted using an antibody against p53. Mouse IgG served as a supershift negative control. **(B)** ChIP assay was performed using an anti-p53 antibody to test whether the p53 protein could bind the IBP promoter in HCT116 p53^+/+^, HCT116 p53^−/−^ and MCF-7 cells. Cells were treated with 50 nmol/L doxorubicin for 8 h. Mouse IgG was used as a negative control.

To further investigate the ability of p53 to bind the putative p53-binding site, 30-bp oligonucleotides that were complementary to the p53-binding site were synthesised, and EMSA was performed using MCF-7 cell nuclear extracts. Nuclear proteins from HCT116 p53^−/−^ were extracted as a negative control. Specific binding was observed in MCF-7 and HCT116 p53^+/+^ cell extracts, but it did not occur in the HCT116 p53^−/−^ extracts. Unlabelled oligonucleotides that were derived from the p53 consensus binding sites of p21 effectively competed with the labelled IBP probe and vice versa. Addition of a p53 antibody to the reaction resulted in a supershift of the labelled bands (Figure 3A). These results demonstrate that p53 specifically binds to p53-binding site of the IBP promoter *in vitro.*

Because p53 protein is able to bind to the IBP promoter *in vitro*, we tested whether p53 can also bind to the IBP promoter in native cellular chromatin. ChIP was performed with a p53 antibody to precipitate chromatin from doxorubicin treated MCF-7, HCT116 p53^−/−^ and HCT116 p53^+/+^ cells (Figure 3B). The precipitated DNA was PCR-amplified using primers that flanked the p53-binding site in the IBP promoter, to produce an expected 156-bp product. When HCT116 p53^+/+^ and MCF-7 cells were treated with 50 nmol/L doxorubicin, the amplified band was increased. This result demonstrates that p53 protein also binds to the IBP promoter p53-binding site *in vivo*. Taken together, these results show that IBP is a direct transcriptional target of p53.

### IBP is suppressed by DNA damaging agents

**Figure 4:**
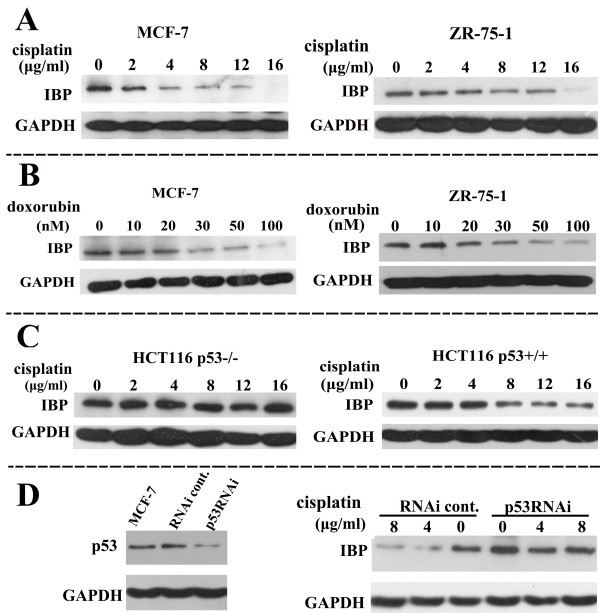
**IBP is p53 dependently suppressed in response to DNA damaging agents.** The expression of IBP was tested with Western analysis. **(A)** MCF-7 and ZR-75-1 which are human breast cancer cells with wild type p53 expression were treated with cisplatin for 24 h. **(B)** MCF-7 and ZR-75-1 cells were treated with doxorubicin for 8 h. **(C)** HCT116 p53^+/+^ cells and HCT116 p53^−/−^ cells were treated with cisplatin for 24 h. **(D)** p53-RNAi MCF-7 cells were treated with cisplatin for different concentrations. Left was the RNAi effect of p53 in MCF-7 cells. GAPDH was used as a control.

Because p53 may be an important mediator of chemotherapeutic toxicity in breast cancer and is induced by DNA damage as a sensor for damaged DNA, we tested whether IBP expression was changed by DNA damaging agents. Cisplatin suppressed IBP expression in a dose-dependent manner in MCF-7 and ZR-75-1 cells that express wild type p53 (Figure 4A). We also detected IBP expression in MCF-7 cells 96h after cisplatin treatment. IBP expression was suppressed by cisplatin in a time-dependent manner within 96h (see Additional file 2). Furthermore, IBP was suppressed with the DNA damaging agent doxorubicin both in MCF-7 and ZR-75-1 cells (Figure 4B). To investigate the p53 dependence of DNA damaging agent-mediated IBP inhibition, we used p53 deleted HCT116 p53^−/−^ cells. IBP was suppressed with cisplatin in HCT116 p53^+/+^ cells, but was unaffected in HCT116 p53^−/−^ cells (Figure 4C). Similar results were obtained in MCF-7 cells stably expressing p53 RNAi (Figure 4D). These data indicate that the suppression of IBP by genotoxic stress in breast cancer cells is p53 dependent.

### IBP regulates the sensitivity to cisplatin-induced apoptosis in MCF-7 cells

**Figure 5:**
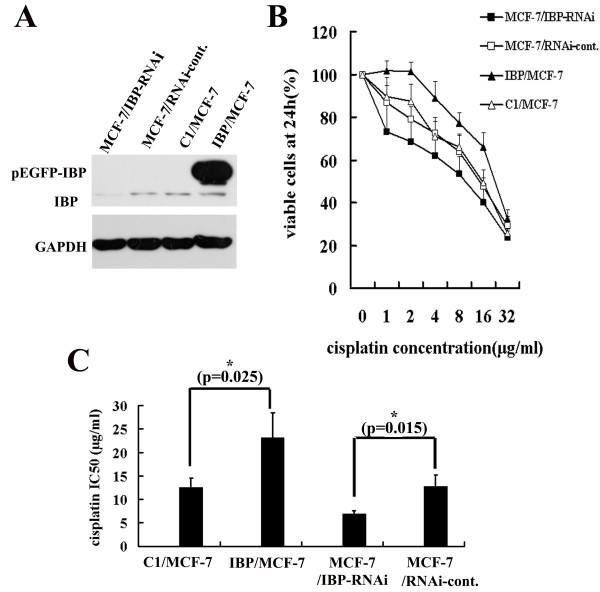
**IBP regulates sensitivity to cisplatin in MCF-7 cells. (A)** IBP expression was analyzed by Western blot in stable IBP-over-expressing MCF-7 cells and IBP-knockdown MCF-7 cells. C1/MCF-7 and MCF-7/RNAi-cont. cells were respective control cells. **(B)** Cells were treated with increasing cisplatin concentrations, and their proliferation rates were measured with a CCK-8 assay. **(C)** Cisplatin IC50 values were determined by CCK cell survival assays. In the columns, the mean was derived from at least three independent experiments. A statistical analysis was performed using Student’s *t* test. *, p < 0.05, significant

It has been shown that p53 pathway is inactive in cisplatin-resistant MCF-7 breast cancer cells [8]. Since IBP is correlated with the malignant behaviour of human breast cancer cells [16] and is down-regulated by p53 and DNA damaging agent in MCF-7 cells, we explored the importance of IBP in the response of MCF-7 to cisplatin. We first established stable IBP over-expressing (IBP/MCF-7) and stable IBP knockdown MCF-7 cells (MCF-7/IBP-RNAi) (Figure 5A). Subsequently, IBP/MCF-7, MCF-7/IBP-RNAi and the corresponding control cells were exposed to cisplatin, and cell growth were measured. Over-expression of IBP increased proliferation and survival of MCF-7 cells, and IBP knockdown increased cisplatin sensitivity of MCF-7 cells (Figure 5B). The IC50 values on IBP knockdown, IBP over-expression, RNAi-control and pEGFP-C1 cells of cisplatin for 24 h were 6.96 ± 0.63 μg/ml, 23.10 ± 5.36 μg/ml, 12.7 ± 2.4 μg/ml and 12.57 ± 1.90 μg/ml, respectively (Figure 5C).

**Figure 6:**
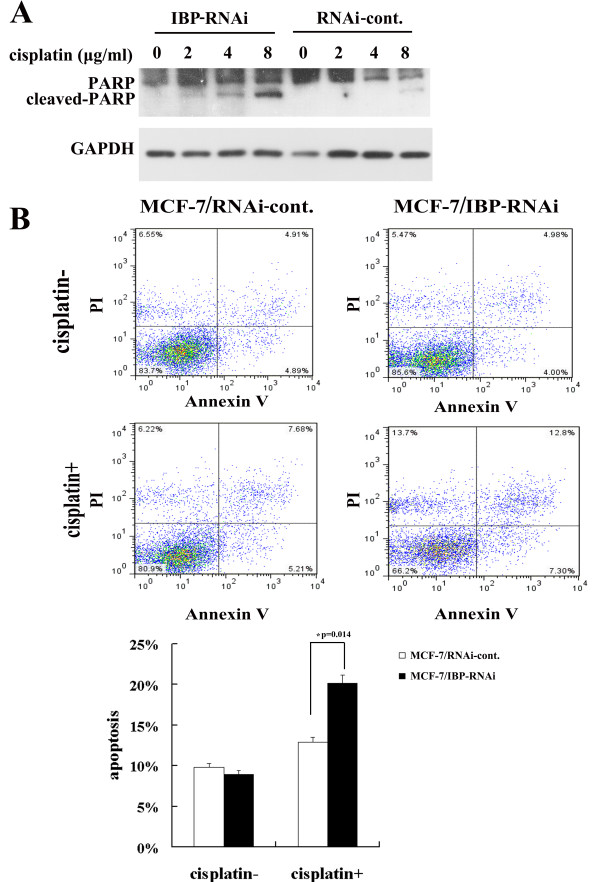
**IBP knockdown results in increased cisplatin-mediated apoptotic cell death. (A)** After a 24 h cisplatin treatment, the cell lysates of the MCF-7/IBP-RNAi cells and the control cells were prepared, and the cytoplasmic marker of apoptosis (cleaved PARP) was detected by western blot. **(B)** MCF-7/IBP-RNAi cells and the control cells were treated with 2 μg/ml cisplatin to induce apoptosis. The apoptosis was detected at 12 h after cisplatin treatment by Alexa fluor 647 conjugated Annexin V-PI assays.

Therefore the decreased survival with cisplatin in MCF-7/IBP-RNAi cells was in large part due to an increase cell death. To confirm that IBP depletion increased cisplatin induced apoptosis in MCF-7 cells, we tested PARP and Annexin V-PI expression. When the cells were treated with cisplatin for 24 h, more cleaved PARP was detected in the MCF-7/IBP-RNAi cells (Figure 6A). In addition, MCF-7/IBP-RNAi cells showed increased percentage of Annexin V-PI positive cells 12 h after cisplatin treatment (Figure 6B). These results demonstrate that IBP participates in the suppression of cisplatin-induced apoptosis in MCF-7 cells.

### IBP over-expression inactivates p53 pathway through AKT

**Figure 7:**
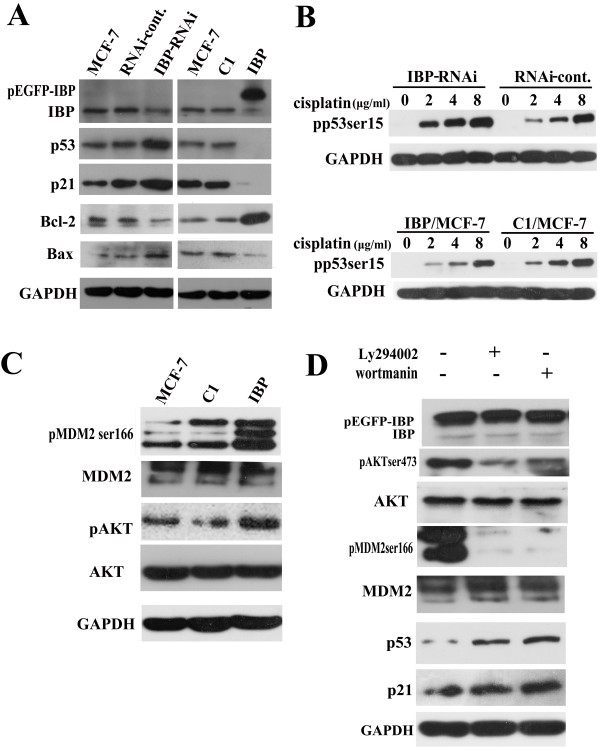
**IBP over-expression activates p53 pathway though AKT. (A)** The relative basal expression levels of IBP, p53, p21, Bcl-2, and Bax in the IBP-knockdown MCF-7 cells (IBP-RNAi) and IBP-over-expressing MCF-7 cells (IBP). **(B)** The expression of p53 Ser-15 phosphorylation was tested in IBP-over-expressing MCF-7 cells and IBP knockdown cells treated with cisplatin for 24 h. **(C)** Western analysis of levels AKT Ser-473, MDM2 Ser-166 phosphorylation in IBP-over-expressing MCF-7 cells and control cells. **(D)** The expression of MDM2 Ser-166 phosphorylation, p53 and p21 were detected in IBP-over-expressing MCF-7 cells treated with Ly294002 or wortmannin for 24 h. GAPDH served as a loading control.

Since IBP suppressed cisplatin induced apoptosis, we further investigated the effect of IBP on cisplatin-induced apoptotic signals. Stabilization and activation of wild type p53 are critical for cisplatin-mediated apoptosis. We tested whether the mechanism of IBP-induced cisplatin resistance was associated with p53 inactivation. Expression of p53 target gene p21 was used to monitor p53 pathway activity. As shown in Figure 7A, the basal expression of p53 in the IBP knockdown MCF-7 cells was markedly elevated. The p21 expression was consistent with p53 expression in IBP-knockdown and IBP-over-expressing MCF-7 cells. Furthermore, we detected cisplatin-induced p53 phosphorylation at Ser-15. In IBP-knockdown cells, increased level of phosphorylated p53 could be induced by cisplatin, whereas lower level p53 Ser-15 phosphorylation was detected in the IBP-over-expressing MCF-7 cells (Figure 7B). This data suggests that IBP over-expression in breast cancer cells decreases p53 accumulation and activation in response to cisplatin.

Members of the Bcl-2 family also are key players in regulating apoptosis. The apoptotic process is regulated by the ratio between Bax and its antiapoptotic counterpart Bcl-2. It is also known that p53 negatively regulates Bcl-2 expression and that wild-type p53 neutralises the death-protective function of Bcl-2 [25,26]. We tested Bcl-2 and Bax levels in IBP-over-expressing MCF-7 cells. The levels of Bcl-2 were highly elevated in IBP-over-expressing MCF-7 cells, and Bax expression was markedly reduced (Figure 7A). This result shows that IBP regulates Bcl-2 family expression, and IBP disruptes p53 dependent apoptotic pathway in breast cancer cells. Thus, there is a positive feedback loop between IBP and p53 pathway.

All p53 auto-regulatory loops are either induced by p53 at the transcriptional level or regulated by p53-induced proteins [27]. It is known that AKT, which is closely associated with DNA damage, induces the phosphorylation of MDM-2 protein, which results in the translocation of MDM-2 into the nucleus where it inactivates p53 [28]. Because the closest homolog of IBP, SWAP-70 [9], is required for the proper activation of AKT [29], we tested whether IBP may also activate AKT. We found high level of AKT Ser-473 and MDM2 Ser-166 phosphorylation in IBP-over-expressing MCF-7 cells (Figure 7C). Moreover, when we treated IBP-over-expressing MCF-7 cells with AKT inhibitor Ly294002 or wortmannin, p53 and p21 expression was elevated, and MDM2 phosphorylation was decreased (Figure 7D). Further, p21 expression in IBP-over-expressing MCF-7 cells treated with Ly294002 or wortmannin for 24 h was quantified (see Additional file 3). These results suggest that IBP may negatively regulate p53 activation through AKT in MCF-7 cells.

### IBP regulates the sensitivity to cisplatin partly through AKT/p53 pathway

**Figure 8:**
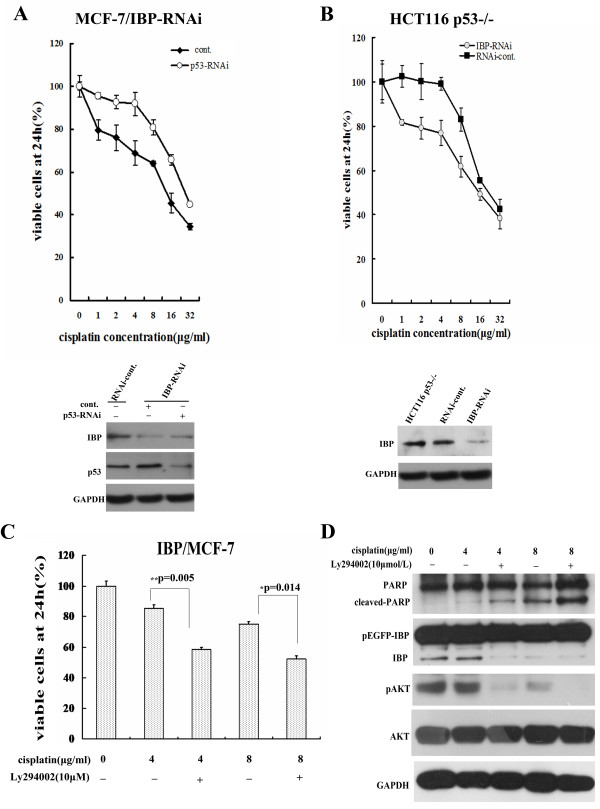
**IBP regulates the sensitivity to cisplatin partly through AKT/p53 pathway. (A)** In stable MCF-7/IBP-RNAi cells, cells were infected with p53 targeting RNAi lentiviral or control lentiviral, and then treated with increasing cisplatin for 24 h. Cell proliferation rates were measured with CCK-8 assay. **(B)** Stable IBP-knockdown HCT116 p53^−/−^ cells were treated with cisplatin for 24 h and cell proliferation were measured. **(C** and **D)** IBP over-expressing MCF-7 cells were treated with cisplatin and 10 μ mol/L Ly294002 for 24 h. Cell proliferation was measured by CCK-8 assay **(C)**. Cleaved PARP and AKT Ser-473 phosphorylation were detected by western blot **(D).**

Since IBP over-expression in turn negatively regulates p53 expression, We further investigated whether IBP regulates the sensitivity to cisplatin in p53-dependent manner. In stable MCF-7/IBP-RNAi cells, we inhibited p53 expression by p53 targeting RNAi lentiviral infection, then cells were exposed to cisplatin, and cell growth was measured. Inhibition of p53 could decrease cisplatin sensitivity in IBP-knockdown MCF-7 cells (Figure 8A). Moreover, we established stable IBP-knockdown HCT116 p53^−/−^ cells, and measured cisplatin-induced cell growth suppression in these cells by using CCK-8. As shown in Figure 8B, IBP knockdown also increased cisplatin sensitivity of HCT116 p53^−/−^ cells. Furthermore, in IBP-over-expressing MCF-7 cells, AKT inhibitors Ly294002 could attenuate cisplatin resistance and increase cisplatin induced apoptosis (Figure 8 C-D). These results suggest that IBP may impair cisplatin chemosensitivity in breast cancer cells partly through AKT/p53 pathway.

## Discussion

IBP is a newly discovered protein aberrantly expressed in breast cancer cells. We found that IBP promotes the proliferation and migration of breast cancer cells and its expression is negatively correlated with p53 levels [16]. Previous studies have shown the role of Lck in IBP activation in T lymphoma cells [12]. However, little is known about the regulation of IBP expression, particularly in breast cancer. Because previous studies have shown that the activity of Rac1 (a downstream molecule of IBP) is inversely regulated by functional p53 [30,31], we investigated whether p53 could regulate IBP in breast cancer cells. Here we have identified IBP as a novel p53 target gene. The inhibition of IBP expression corresponded with increased p53 expression, and the induction of IBP was related to p53. p53 could bind to IBP promoter in MCF-7 cells. The present results clearly indicate that inactivation of wild-type p53 at least partially explains the aberrant IBP expression in breast cancer.

It was previously reported that p53 could transactivate genes from a noncanonical consensus 1/2-site or 3/4-sites that contain a 1/4-site that is adjacent to a 1/2-site or a 1/4-site and is separated from a 1/2-site by a 5-nt spacer [32]. We have shown for the first time that IBP promoter region possesses a noncanonical repressing p53-binding site. We identified that IBP promoter contains a “perfect” p53 half site, which contains a CATG core motif. It is known that the C and G positions are essential for the function of the p53-binding site, and the presence of an “AT” as the “WW” dinucleotide is associated with the high activity of a half site [2,33]. Ren’s group reported that CATG core was an activating core, but the nucleotides adjacent to the CWWG motif could modulate p53 function to become repressive, and repressing p53 response elements had a much higher frequency of noncanonical nucleotides in the position immediately adjacent to the CWWG motif [34]. The triplet flanking sequences in the p53-binding site of IBP promoter also differ from the canonical p53-binding site motif. However, whether the triplet flanking sequences in the half p53-binding site or the 1/4-site that is adjacent to a 1/2-site modulate the p53 response element behaviour in IBP promoter, needs further investigation. In addition, it has been shown that p53 mutants can also transactivate gene expression at noncanonical sites [32,33]. Noncanonical sequences may exhibit responsiveness to p53 in combination with other transcription factors, such as the estrogen receptor [33]. In this study, although the role of the p53 mutants or the possible cofactors in IBP transcription in breast cancer remains to be determined, further experiments will elucidate the mechanism of aberrant IBP expression in breast cancer cells.

So far little information is available concerning the function of IBP, especially in breast cancer. IBP is a GEF related to the Rho GTPases. Recent study showed a new function for GEFs in the modulation of cell death after genotoxic stress [35]. It is also reported that Cdc42 activity downstream of IBP might regulate mammalian genomic stability [36]. In the present study, we have shown that IBP is decreased upon exposure to DNA damaging agents in a p53 dependent manner. It is known that the status of p53 is associated with resistance to DNA-damaging therapies [37,38]. p53 mutations are common in breast cancer cells and p53 inactivation is an important cause for cisplatin resistance [8]. p53 pathway plays an important role in DNA damage mediated apoptotic signals. Here we further demonstrated that IBP regulated cisplatin-mediated apoptosis in MCF-7 cells. IBP over-expression increased cisplatin resistance in MCF-7 cells. The response to DNA damaging agent and the mechanisms of cisplatin resistance are complex and multifactorial. It is likely that IBP is one of the mediators for a p53-dependent cisplatin response in breast cancer cells. Mechanisms that inhibit the propagation of DNA damage signalling to the apoptotic machinery are complex. We found that IBP over-expression in MCF-7 cells suppressed the basal protein expression of p53 and p21, attenuated p53 phosphorylation, changed the ratio between Bax and Bcl-2, and activated AKT. It is known that in chemoresistant cells cisplatin induced p53 phosphorylation is attenuated, particularly on Ser15 and Ser20, and the phosphorylation of Ser15 and Ser20 plays an important role in the transduction of p53-mediated apoptosis [39]. These results indicate that IBP plays a role in increased cisplatin resistance in at least three aspects: the loss of p53 function, over-expression of antiapoptotic Bcl-2, and activation of the PI3K/AKT pathway. Although our data explained in partly the mechanisms of IBP-mediated suppression of breast cancer cell apoptosis in response to cisplatin, whether this function is related to RhoGTPase (e.g. Cdc42) is still unknown. Other study has shown that p53-mediated reactive oxygen species (ROS) production could also be a mechanism of cisplatin-induced apoptosis [40]. It is clear that Rac1 is an important regulator of ROS production [41,42]. Whether IBP regulates cisplatin resistance through Rac1 and ROS remains to be confirmed. In addition, it is interesting that our results also suggest that IBP over-expression in breast cancer cells may possibly induce a potential p53 regulatory feedback loop.

## Conclusions

In summary, we provide evidence that IBP, which is a direct target gene of p53, is inversely regulated by p53. We observed that IBP over-expression decreases cisplatin-mediated breast cancer cell apoptosis, while IBP suppression reduces cisplatin resistance. We also observed that IBP is a feedback regulator of p53. These observations promote our understanding of the relationship between IBP signalling and the p53 tumour suppressor. Therefore IBP may serve as a target for pharmacologic intervention of breast cancer resistant to cisplatin therapy.

## Materials and methods

### Cell lines

HEK293 cells and human breast cancer MCF-7 cells, ZR-75-1 cells, were purchased from the Type Culture Collection of the Chinese Academy of Sciences (Shanghai, China). The HCT116 p53^−/−^ and HCT116 p53^+/+^ cell lines were gifts from Dr. Vogelstein (Johns Hopkins University, USA) [43] and Dr. Zhihua Liu (Chinese Academy of Medical Sciences and Peking Union Medical College, China) [44]. MCF-7 cells were grown in MEM medium that was supplemented with 10% foetal bovine serum, 1% non-essential amino acids and 10 μg/ml insulin. ZR-75-1 cells were grown in RPMI-1640 medium with 10% foetal bovine serum. HEK293 cells, HCT116 p53^−/−^ and p53^+/+^ cells were maintained in DMEM that was supplemented with 10% foetal bovine serum. All of the cells were maintained in a humidified atmosphere that contained 5% CO_2_ at 37°C.

### Plasmid construction and mutagenesis

The −1807/+60, −979/+60, −640/+60, −294/+60 and −116/+60 fragments of the human IBP gene (relative to the transcriptional start site) were amplified from the genomic DNA of MCF-7 cells by PCR using KOD polymerase (Toyobo). These amplified fragments were inserted into the *Kpn*I and *Hind*III restriction sites of the pGL3-basic vector (Promega). The wild-type p53 expression plasmid, pCMV-p53, and the p53 mutant plasmid, pCMV-p53R175H, were kindly provided by Dr. Vogelstein (Johns Hopkins University, USA). TaKaRa MutanBEST kit (TaKaRa) was used to introduce the p53 binding site into the IBP promoter deletion mutant. The following mutagenic primers were used: forward 5′-CGGGAGCCACGTGGATACAG-3′, reverse 5′-TTTTAGAAGCCTCCTCAGACCC-3′. The pEGFP-C1-IBP expression plasmid was a gift from Dr. Alessandra B. Pernis (Columbia University, USA). All of the constructs were confirmed by DNA sequencing.

### Adenovirus infection and cell treatment

Adenovirus(Ad)-p53 was purchased from Shenzhen SiBiono GeneTech Co. [44]. Ad-GFP was purchased from Shanghai Sunbio Medical Biotechnology Co. The cells were treated with different concentrations of doxorubicin (Sigma-Aldrich) for 8 h, Nutlin-3 (Beyotime) for 24 h and pifithrin-α (Beyotime) for 24 h. The cisplatin (Sigma-Aldrich) concentrations and experimental details are described in the text and figure legends. The cells were treated with Ly294002 (Beyotime) or wortmannin (Beyotime) for 24 h.

### RNA interference

To knockdown IBP expression, double-stranded DNA oligonucleotides (forward, 5′-TGCTGTTCATCTGGACATTCCAGTGTGTTTTGGCCACTGACTGACACACTGGAGTCCAGATGAA-3′ and reverse, 5′-CCTGTTCATCTGGACTCCAGTGTGTCAGTCAGTGGCCAAAACACACTGGAATGTCCAGATGAAC-3′) were subcloned into pcDNA™6.2-GW/EmGFPmiR (Invitrogen) using the BLOCK-iT Pol II miR RNAi Expression Vector Kit (Invitrogen). The RNAi plasmid or control plasmid, which contained a non-specific sequence, was transfected into MCF-7 cells. Lipofectamine 2000 (Invitrogen) was used as the transfection reagent. The growth medium was supplemented with blasticidin (10 μg/ml, Invitrogen), which was used to select for blasticidin-resistant transfectants. For the p53 knockdown, double-stranded DNA oligonucleotides (forward, 5′-CCGGGACTCCAGTGGTAATCTACTTCAAGAGAGTAGATTACCACTGGAGTCTTTTTG-3′ and reverse, 5′-AATTCAAAAAGACTCCAGTGGTAATCTACTCTCTTGAAGTAGATTACCACTGGAGTC-3′) were subcloned into pMagic 1.1 and packaged into lentivirus particles (Shanghai Sunbio Medical Biotechnology Co.). One day after infection, the cell-growth medium was supplemented with puromycin (2 μg/ml, Invitrogen) to select stable transfectants.

### Luciferase reporter assays

Luciferase reporter assays were performed using the Dual-*Luciferase*® Reporter Assay System (Promega). Cells were seeded in 24-well plates (1.0 × 10^5^ cells/well) and transfected together with a promoter-reporter gene vector and the pRL-TK *Renilla* luciferase vector. After 48 h of transfection, the cells were harvested and analysed according to the manufacturer’s instructions. The luciferase activities were normalised to the *Renilla* luciferase activity of the internal control.

### Western blotting

Cell lysates were prepared in RIPA buffer (Beyotime). Whole-cell lysates were separated on a 10% SDS-PAGE gel and transferred onto polyvinylidene difluoride (PVDF) membranes (Millipore). The membranes were blocked for 1 h at 37°C in 5% non-fat milk/TBST and were then incubated with primary antibodies overnight at 4°C. Antibodies against IBP (produced as described previously) [16], p53 (sc-126, Santa Cruz), p21 (3733–1, Epitomics), PARP (9532, Cell Signaling), phospho-p53(Ser15) (9284, Cell Signaling), Bcl-2 (1017–1, Epitomics), Bax (AB026, Beyotime), phospho-AKT(Ser473) (4060, Cell Signaling), AKT(4691, Cell Signaling),phospho-MDM2(Ser166) (3521, Cell Signaling), MDM2(sc-965, Santa Cruz) and GAPDH (AG019, Beyotime) were used. The membrane was then rinsed in TBST and incubated with various secondary antibodies for 2 h at 25°C. Immunoreactive bands were visualised with a chemiluminescent HRP substrate (Millipore).

### Quantitative RT-PCR

Total RNA was isolated using TRIzol (Invitrogen), and 1 μg of isolated RNA was reverse transcribed to generate cDNAs (TaKaRa). Amplification was performed by using SYBR Premix Ex Taq II (TaKaRa). The primers used for amplification included the following: IBP forward, 5′-GAGGGCTGACGAGGATGTGG-3′ and reverse, 5′-GCTGGTGACCGGACGCTTAT-3′; and GAPDH forward, 5′-AATCCATCACCATCTTCCA-3′ and reverse, 5′-TGGACTCCACGACGTACTCA-3′. GAPDH mRNA levels were determined as an internal control.

### Electrophoretic mobility shift assays (EMSA)

Nuclear extracts were prepared in hypertonic buffer (420 mM NaCl, 1.5 mM MgCl_2_, 0.5 mM DTT, 0.2 mM EDTA, 0.5 mM PMSF, 25% glycerol, 5 μg/ml aprotinin, 5 μg/ml phenanthroline, 3 μg/ml pepstatin A and 20 mM HEPES). Double-stranded oligonucleotide probes that were derived from the IBP gene promoter (sense strand, 5′-TAAAAGGGCCTCACATGCCCCGGGAGCCAC-3′) and *p21* gene promoter (sense strand, 5′-GGAAGAAGACTGGGCATGTCTGGGCAGAGA-3′) [44] were labelled with γ-^32^P-ATP using T4 polynucleotide kinase. The nuclear extracts (8 μg) were incubated with the probe for 30 min at 30°C. The protein-DNA complexes were resolved using non-denaturing PAGE and were detected by autoradiography. For the cold probe competition assay, unlabelled probe was added to the nuclear protein extracts one hour before the detection was performed. In the supershift assay, 1 μl of an anti-p53 antibody (sc-126x, Santa Cruz) was incubated with the nuclear extracts for 1 h at room temperature prior to the addition of the radiolabeled probe and the implementation of PAGE.

### Chromatin immunoprecipitation assay (ChIP)

The ChIP assays were performed using an EZ-ChIP^TM^ Chromatin Immunoprecipitation Kit (Upstate) following the manufacturer’s instructions. Briefly, cells were crosslinked with 1% formaldehyde and a p53 antibody (sc-126, Santa Cruz) or control IgG, which was used to precipitate the crosslinked protein/chromatin. The DNA fragments were analysed using PCR with a primer set (forward, 5′-TTTTCCCTCAGCAAGCTGCGTCTGG-3′ and reverse, 5′-CTGCATGGGAACTGGGGACCAACTCT-3′) that was designed to amplify the −305 to −150 region of the IBP gene that harbours p53-binding site.

### Cell survival assays

A cell survival assay was performed in triplicate with a Cell Counting Kit-8 (CCK-8, Beyotime). The cells were seeded in 96-well plates at 5 × 10^3^ cells/well (100 μl/well) 24 h before the cisplatin treatment. The culture medium was then replaced with fresh medium that contained different concentrations of cisplatin, which ranged from 0 to 32 μg/ml, and the cells were cultured in this medium for 24 h. Following the incubation, 10 μl of CCK-8 solution was added to each well, and after 1 h, the absorbance value of each well was read at 450 nm. The cell growth rate was calculated as the ratio of the absorbance of the experimental well to that of the blank well. The IC50 values (the drug concentration that results in a 50% absorbance reduction compared to the control) were calculated.

### Annexin V-PI flow cytometry assay

Flow cytometry assay was performed by using Caliber II sorter and Cell Quest FACS system (BD Biosciences). Alexa fluor 647 conjugated Annexin V (invitrogen) and PI (Invitrogen) was incubated for 15 min according to the manufacturer’s protocol. About 10^4^ cells were measured per sample.

## Abbreviations

Ad, Adenovirus; ChIP, Chromatin immunoprecipitation; EMSA, Electrophoretic mobility-shift assay; GEF, Guanine nucleotide exchange factor; GFP, Green fluorescent protein; IBP, Interferon regulatory factor 4 binding protein; PARP, Poly-ADP ribose polymerase; RNAi, RNA interference.

## Competing interests

The authors declare that they have no competing of interests.

## Authors’ contributions

MY and CH designed the study. MY, FY and ZC performed the experiments and analysed and interpreted the results. PL and AC provided technical and material support. MY wrote the manuscript. PL and SL revised the manuscript. CH supervised the study. All authors read and approved the final manuscript.

## Supplementary Material

Additional file 1 **Figure S1.** In HCT116 p53^−/−^ cells, Nutlin-3 could not decrease IBP expression. IBP expression was detected by western blot when HCT116 p53^−/−^ cells were treated with different concentration of Nutlin-3 for 8 h. Click here for file

Additional file 2 **Figure S2.** IBP expression is not increase in response to cisplatin within 96 h in MCF-7 cells. IBP and p53 expression was detected by western blot when MCF-7 cells were treated with 8 μg/ml cisplatin continuously for 12 h to 96 h. Click here for file

Additional file 3 **Figure S3.** Quantification analysis for p21 expression in IBP-over-expressing MCF-7 cells treated with Ly294002 or wortmannin for 24 h. Click here for file
