# Supplementary figures and images for: Interferon regulatory factor 4 binding protein is a novel p53 target gene and suppresses cisplatin-induced apoptosis of breast cancer cells
Source: Mol Cancer. 2012 Aug 13;11:54. doi: 10.1186/1476-4598-11-54 (PMC3447665; doi:10.1186/1476-4598-11-54)

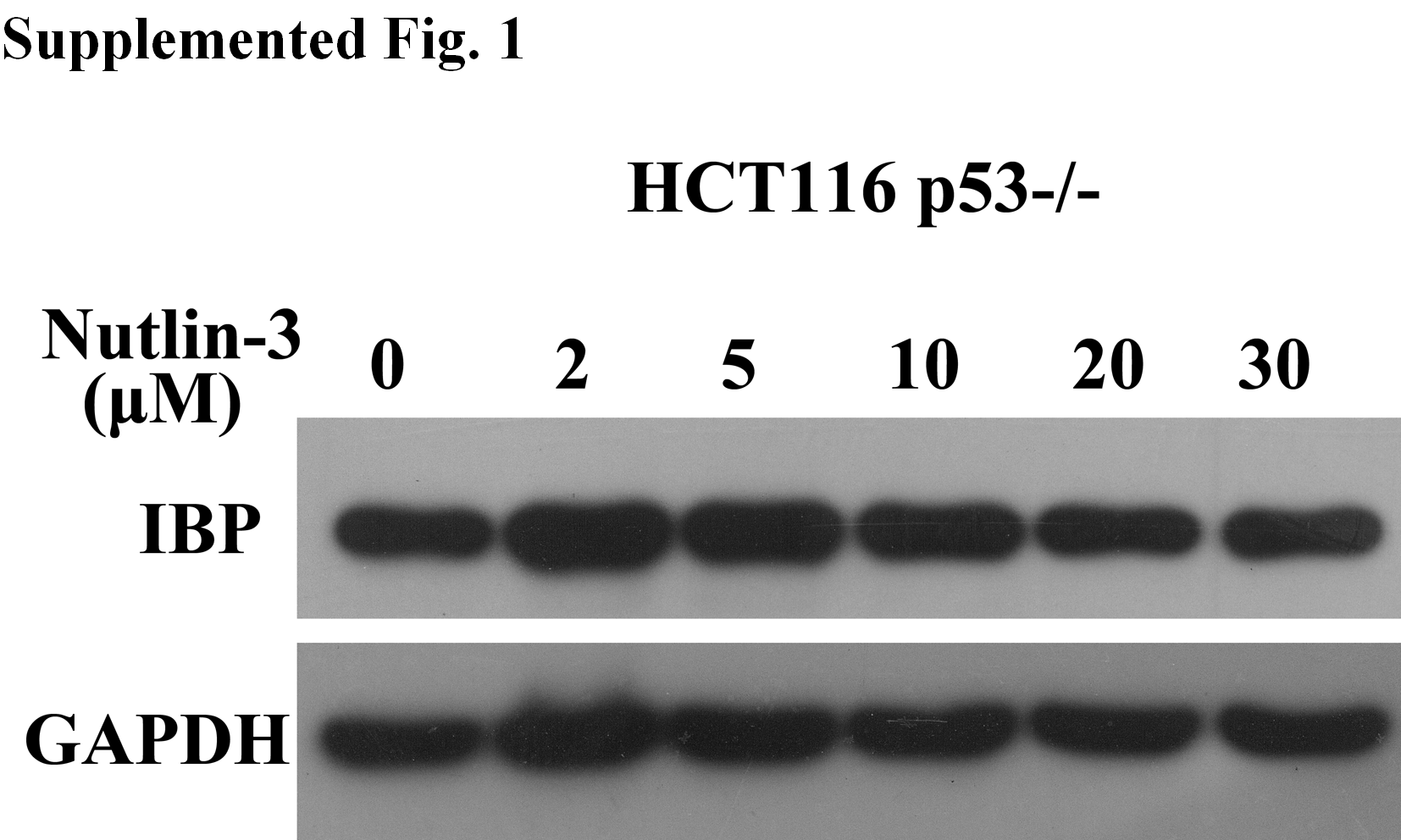

Supplement: Additional file 1 — Figure S1. In HCT116 p53−/− cells, Nutlin-3 could not decrease IBP expression. IBP expression was detected by western blot when HCT116 p53−/− cells were treated with different concentration of Nutlin-3 for 8 h. [file 1476-4598-11-54-S1.tiff]

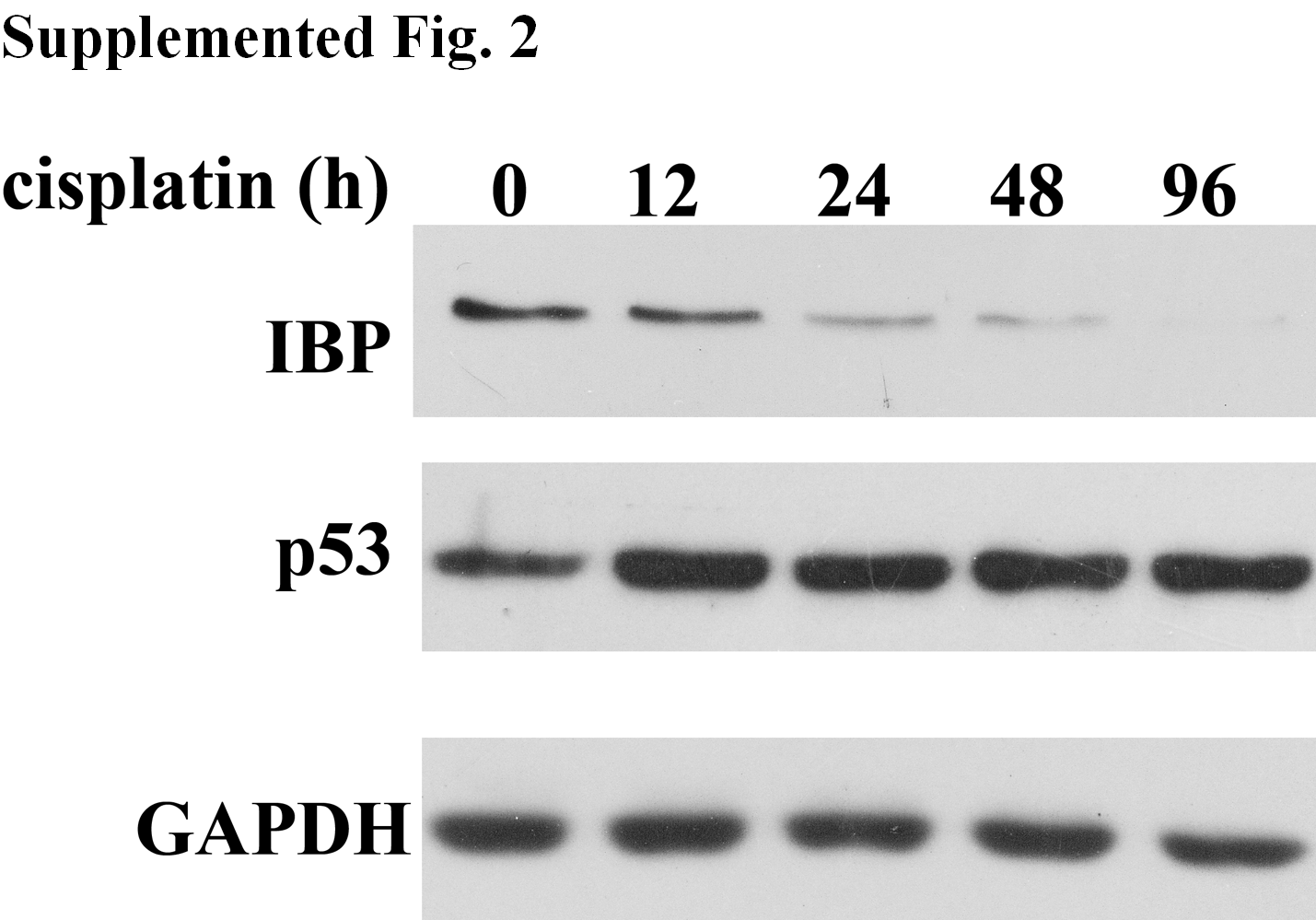

Supplement: Additional file 2 — Figure S2. IBP expression is not increase in response to cisplatin within 96 h in MCF-7 cells. IBP and p53 expression was detected by western blot when MCF-7 cells were treated with 8 μg/ml cisplatin continuously for 12 h to 96 h. [file 1476-4598-11-54-S2.tiff]

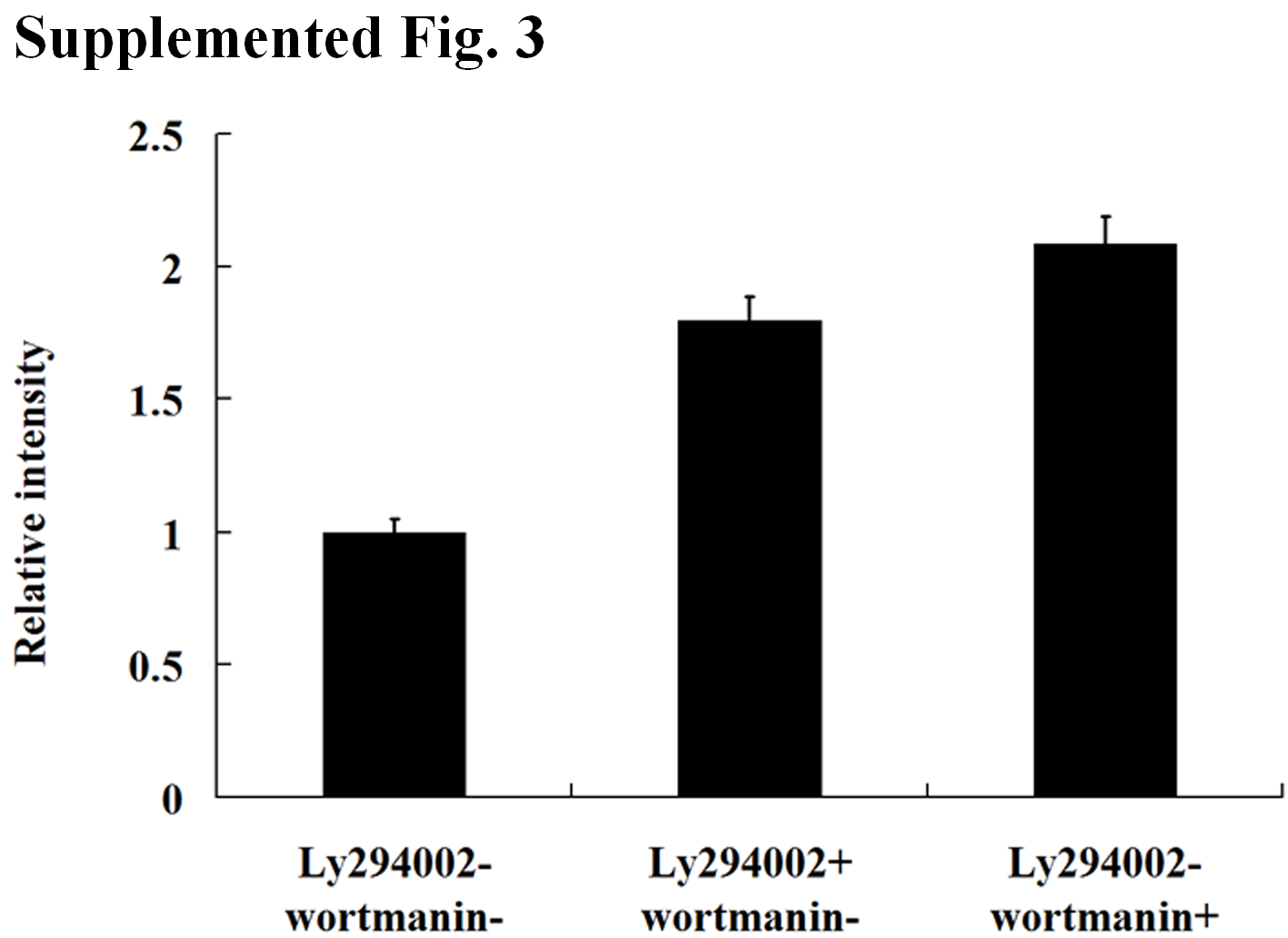

Supplement: Additional file 3 — Figure S3. Quantification analysis for p21 expression in IBP-over-expressing MCF-7 cells treated with Ly294002 or wortmannin for 24 h. [file 1476-4598-11-54-S3.tiff]
